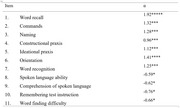# Tasks discriminating cognitive impairment levels: Item Response Theory applied to the Alzheimer's Disease Assessment Scale Cognitive Subscale

**DOI:** 10.1002/alz70857_096753

**Published:** 2025-12-24

**Authors:** Anat Rotstein, Stephen Z Levine, Yair Goldberg, Myrto Samara, Kazufumi Yoshida, Andrea Cipriani, Takeshi Iwatsubo, Stefan Leucht, Toshiaki A Furawaka

**Affiliations:** ^1^ University of Haifa, Haifa, Israel; ^2^ Technion‐ Institute of Technology, Haifa, Israel; ^3^ Technical University of Munich, Munich, Germany; ^4^ Department of Health Promotion and Human Behavior, Graduate School of Medicine/ School of Public Health, Kyoto University, Kyoto, Japan; ^5^ University of Oxford, Oxford, United Kingdom; ^6^ Department of Neuropathology, Graduate School of Medicine, The University of Tokyo, Bunkyo‐ku, Tokyo, Japan; ^7^ Unit for Early and Exploratory Development, The University of Tokyo, Tokyo, Japan; ^8^ National Center of Neurology and Psychiatry, Tokyo, Japan; ^9^ Technische Universität München, Munich, Germany; ^10^ Kyoto University School of Public Health, Kyoto, Japan

## Abstract

**Background:**

Since cognitive impairment is a clinical hallmark of Alzheimer's disease, suitable assessments are essential for treatment and research following onset. The most widely used and researched cognitive impairment measure in clinical trials of Alzheimer's Disease is the Alzheimer's Disease Assessment Scale Cognitive Subscale. Early evidence reported that the measure demonstrated acceptable levels of reliability and validity based on traditional psychometric approaches. Later, more advanced psychometric approaches, such as Item Response Theory, have been implemented to evaluate this measure. Item Response Theory provides details on reliability of different cognitive impairment levels for each item. We aimed to examine tasks discriminating cognitive impairment levels using Item Response Theory based on individual‐level participant clinical trial data.

**Method:**

*Participants*: Individual‐level participant data (*N* = 2,198) of five randomized controlled double‐blinded trials of donepezil conducted by Eisai Co. Ltd.

*Measures: Alzheimer*'*s Disease Assessment Scale Cognitive Subscale* is a neuropsychological index of cognitive impairment, indicating the severity of cognitive symptoms in Alzheimer's disease. It consists of 11 items to assess memory, language, and praxis functions.

*Analytic plan*: The Graded Response Model, a form of Item Response Theory, was implemented in the 'ltm' package in R. Item discrimination parameters (α) map the ability of an item to discriminate impairment levels. Discrimination parameter values for items ranging from 0.01 to 0.24 are considered very low, 0.25 to 0.64 are low, 0.65 to 1.34 are moderate, 1.35 to 1.69 are high, and over 1.7 are very high.

**Result:**

Word recall had the highest ability to discriminate underlying cognitive impairment levels (α=1.92). Four tasks (Spoken language ability, Comprehension of spoken language, Remembering test instruction, and Word finding difficulty) had low item discrimination parameters meaning that these tasks lacked the ability to discriminate underlying cognitive impairment levels. This left seven discriminating tasks (Word recall, Commands, Naming, Constructional praxis, Ideation praxis, Orientation, Word recognition).

**Conclusion:**

Seven items separated placebo from donepezil in five pivotal clinical trials of donepezil compared with placebo for Alzheimer's disease. The current study contributes to knowledge on Alzheimer's disease by identifying tasks that discriminate cognitive impairment levels, with potential use for treatment monitoring in moderate‐stage Alzheimer's disease.